# Four-year follow-up of LCAR-B38M in relapsed or refractory multiple myeloma: a phase 1, single-arm, open-label, multicenter study in China (LEGEND-2)

**DOI:** 10.1186/s13045-022-01301-8

**Published:** 2022-07-06

**Authors:** Wan-Hong Zhao, Bai-Yan Wang, Li-Juan Chen, Wei-Jun Fu, Jie Xu, Jie Liu, Shi-Wei Jin, Yin-Xia Chen, Xing-Mei Cao, Yun Yang, Yi-Lin Zhang, Fang-Xia Wang, Peng-Yu Zhang, Bo Lei, Liu-Fang Gu, Jian-Li Wang, Hui Zhang, Ju Bai, Yan Xu, Han Zhu, Juan Du, Hua Jiang, Xiao-Hu Fan, Jian-Yong Li, Jian Hou, Zhu Chen, Wang-Gang Zhang, Jian-Qing Mi, Sai-Juan Chen, Ai-Li He

**Affiliations:** 1 Department of Hematology, The Second Affiliated Hospital of Xi’an Jiaotong University, 157 West 5th Road, Xi’an, 710004 China; 2 Department of Hematology, Jiangsu Province Hospital, First Affiliated Hospital of Nanjing Medical University, Nanjing, 210029 China; 3 Department of Hematology, Changzheng Hospital, The Second Military Medical University, Shanghai, 200003 China; 4 Department of Hematology, School of Medicine, Shanghai Fourth People’s Hospital, Tongji University, Shanghai, 200434 China; 5 State Key Laboratory of Medical Genomics, National Research Center for Translational Medicine, Shanghai Institute of Hematology, Ruijin Hospital Affiliated With Shanghai Jiao Tong University School of Medicine, 197 Rui Jin er Road, Shanghai, 200025 China; 6 Nanjing Legend Biotech Inc., Nanjing, 210000 China; 7 Department of Hematology, Renji Hospital Affiliated With Shanghai Jiao Tong University School of Medicine, Shanghai, 200127 China; 8 Department of Hematology and National-Local Joint Engineering Research Center of Biodiagnostics and Biotherapy, The Second Affiliated Hospital of Xi’an Jiaotong University, Xi’an, China

**Keywords:** Multiple myeloma, Chimeric antigen receptor therapy, B cell maturation antigen, Safety, Efficacy

## Abstract

**Background:**

LCAR-B38M is a chimeric antigen receptor T cell product with two binding domains targeting B cell maturation antigen. Our previous reports showed a remarkable efficacy of LCAR-B38M in patients with relapsed/refractory multiple myeloma (RRMM) at a median follow-up of 2 years. Here, we report long-term safety and efficacy data from a median follow-up of 4 years.

**Methods:**

LEGEND-2 was a phase 1, single-arm, open-label study conducted in four registered sites in China. Seventy-four participants with RRMM received LCAR-B38M treatment. Lymphodepletion was performed using cyclophosphamide or cyclophosphamide plus fludarabine. LCAR-B38M, at a median dose of 0.513 × 10^6^ cells/kg, was intravenously administered either in three split infusions or in a single infusion. The primary objective was the safety of LCAR-B38M, and the secondary objective was efficacy.

**Results:**

As of May 25, 2021, the median follow-up was 47.8 months. All patients experienced ≥ 1 adverse events (AEs). Grade ≥ 3 AEs were observed in 45/74 (60.8%) patients. Cytokine release syndrome (CRS) occurred in 68/74 (91.9%) cases; 7 (9.5%) had grade ≥ 3 CRS. One patient experienced grade 1 central nervous system toxicity. The overall response rate was 87.8%. Fifty-four out of 74 (73.0%) patients achieved complete response. The median progression-free survival was 18.0 months, and the median overall survival for all patients was not reached. The median duration of response was 23.3 months. Four patients experienced viral infection more than 6 months post-infusion, and four patients developed second primary non-hematological malignancies at a median time of 11.5 months post-CAR-T cell transfer.

**Conclusions:**

The 4-year follow-up data of LCAR-B38M therapy demonstrated a favorable long-term safety profile and a durable response in patients with RRMM.

*Trial registration* Clinicaltrials.gov NCT03090659 (retrospectively registered on March 27, 2017); ChiCTR-ONH-17012285.

**Supplementary Information:**

The online version contains supplementary material available at 10.1186/s13045-022-01301-8.

## Background

Multiple myeloma (MM), the second most common hematological malignancy, is characterized by malignant terminally differentiated plasma cells. It preferentially affects individuals aged over 60 years, complicating treatment with chemotherapies. In the era of conventional chemotherapy, the 5-year overall survival (OS) was less than 30%. Over the past three decades, the advent of novel agents, including proteasome inhibitors (PIs), immunomodulatory drugs (IMiDs), and anti-CD38 monoclonal antibodies, has improved therapeutic response and prolonged survival [1–3]. However, most patients still face relapse and/or drug resistance, necessitating the development of a new therapy with a novel mechanism of action.

Encouragingly, chimeric antigen receptor (CAR)-reprogrammed autologous T cell treatment holds promise. Initial success came from CAR-T cells targeting CD19 to treat relapsed/refractory (RR) hematological B cell malignancies, proving that the CAR platform can eradicate cancerous cells by targeting tumor surface antigens [4–7]. B cell maturation antigen (BCMA) is a cell surface glycoprotein belonging to the tumor necrosis factor superfamily. It is exclusively expressed by B-lineage cells and is essential for plasma cell survival; thus, it is in theory an ideal target for the treatment of MM [4, 8–10].

The LEGEND-2 study (NCT03090659, ChiCTR-ONH-17012285) was a first-in-human trial of CAR-T cell therapy in RRMM. The LCAR-B38M lentiviral construct encodes a llama-derived, tandem heavy chain-based CAR consisting of two BCMA-targeting single-domain antibodies designed to confer avidity, and a 4-1BB/CD3ζ intracellular signaling domain. In total, 74 patients with RRMM were enrolled. Fifty-seven patients were treated at the Second Affiliated Hospital of Xi’an Jiaotong University, where lymphodepletion using cyclophosphamide and three-split infusion was carried out [11, 12]. The remaining 17 patients received LCAR-B38M treatment at a geographically separate region (East China) with three registered centers: Ruijin Hospital affiliated with Shanghai Jiao Tong University, First Affiliated Hospital of Nanjing Medical University, and Changzheng Hospital. At these sites, fludarabine + cyclophosphamide-based conditioning therapy combined with three-split infusion or cyclophosphamide combined with one-dose infusion was performed [13]. Initial results from the LEGEND-2 study showed deep, durable responses with manageable safety in patients heavily pretreated with standard therapies, including PIs, IMiDs, or autologous hematopoietic stem cell transplantation. Regionally independent analyses yielded the same overall response rate (ORR) of 88%. With a median follow-up time of 2 years, the patients at the Xi’an site achieved a median progression-free survival (PFS) of 19.9 months and the patients at the other three sites in East China reached 18.0 months [11–13].

Subsequently, the CARTITUDE-1 study, a phase 1b/2 evaluation of LCAR-B38M CAR-T cells (known as ciltacabtagene autoleucel [cilta-cel] in this study), adopted the strategy of fludarabine + cyclophosphamide-based lymphodepletion and a single infusion of CAR-T cells in heavily pretreated patients with RRMM in the USA and Japan. At a median 22 months of follow-up, CARTITUDE-1 had an ORR of 98% and median PFS and median OS were not reached [14–16], confirming the therapeutic potential of LCAR-B38M in the treatment of RRMM.

Herein, we provide 4-year follow-up data on all 74 patients treated with LCAR-B38M in the LEGEND-2 study. This represents the longest follow-up of CAR-T therapy in RRMM to date.

## Methods

### Study design and treatment

LEGEND-2 (NCT03090659) was a phase 1, single-arm, open-label study conducted in four centers in China. LCAR-B38M is an engineered autologous T cell transduced with a CAR plasmid via lentivirus transfer system. The functional elements of the CAR construct contain two BCMA-targeting single-domain antibodies, CD8a hinge and transmembrane domain, and 4-1BB/CD3ζ intracellular signaling domain. The study design and clinical management detailed in all study sites have been previously published [12, 13]. In brief, enrolled patients were aged 18–80 years and met the inclusion criteria of RRMM. Each study site had its own protocol for lymphodepletion and timing of CAR-T cell administration. Lymphodepletion was performed using cyclophosphamide 300 mg/m^2^ or cyclophosphamide 250 mg/m^2^ plus fludarabine 25 mg/m^2^ (Cyc + Flu); LCAR-B38M CAR-T cells were infused either in three separate infusions (65 patients) or in a single infusion (9 patients). An institutional review board or independent ethics committee at each study site approved the study protocol, and all patients provided informed consent.

### Long-term outcome assessments

Adverse events (AEs) were graded using National Cancer Institute Common Terminology Criteria for Adverse Events version 4.03. Cytokine release syndrome (CRS) was assessed using the modified criteria proposed by Lee et al. [17]. Any virus infection, including herpes zoster virus infection and hepatitis B virus activation, was recorded after infusion. Due to the prevalence of hepatitis B virus infection in China, a negative hepatitis B virus quantitative DNA test was required before enrollment. In addition, the occurrence of second primary malignancies was recorded during follow-up.

Efficacy was assessed by the criteria of response according to International Myeloma Working Group consensus recommendations [18], including ORR, duration of response (DOR) for responders, OS, and PFS. Complete response evaluation in patients with extramedullary disease (EMD) included bone marrow aspirate, blood and urine M protein, and imaging. Minimal residual disease (MRD) was assessed at a sensitivity threshold of 10^–4^ by two 8-color flow cytometry panels: CD38, CD45, CD19, CD56, CD27, CD81, CD200, CD20; and CD38, CD138, CD19, CD45, CD117, CD28, cytoplasmic kappa, and cytoplasmic lambda. The correlation of soluble BCMA (sBCMA), antidrug antibodies (ADAs), lymphodepletion regimen, and pharmacokinetic profile with clinical outcome was also analyzed.

### Statistical analysis

Efficacy and safety analyses were conducted in all patients who received at least 1 dose of LCAR-B38M CAR-T cells. ORR was defined as the proportion of patients who achieved a partial response (PR) or better. Two-sided 95% exact confidence intervals (CIs) based on binomial distribution were calculated for each response category. Median DOR, PFS, and OS and corresponding 95% CIs were calculated using Kaplan–Meier methods. Clinical response comparison among different biomarker groups was made by a log-rank test, abnormal data comparison was made by Wilcoxon rank-sum test, and a chi-square test was applied for comparing proportions of a categorical outcome among different groups.

## Results

### Patient characteristics

This phase 1 clinical trial enrolled patients from March 30, 2016, to November 26, 2017. Seventy-four patients underwent leukapheresis (Xi’an site, *n* = 57; Rujin site, *n* = 5; Changzhen site, *n* = 3; Jiangsu site, *n* = 9); preconditioning lymphodepletion regimens and infusion doses are shown in Additional file 1: Fig. S1. Median patient age was 54.5 years (range, 27–74), and 60.8% were male (Table 1). The median number of prior lines of therapy was 3 (range, 1–9). Most patients had prior exposure to a PI (73.0%) or IMiD (87.8%), and 64.9% were exposed to both; 24.3% of patients had relapsed after autologous stem cell transplantation. Baseline disease was International Staging System stage III in 28.4% of patients, and 29.7% had extramedullary involvement.

**Table 1:** Baseline characteristics

Characteristic	Total (*N* = 74)
Age, median (range), yr	54.5 (27–74)
Male sex, *n* (%)	45 (60.8)
Time since diagnosis, median (range), yr	4.0 (1–9)
ECOG performance status score, *n* (%)	
0	30 (40.5)
1	32 (43.2)
2	12 (16.2)
ISS stage, *n* (%)	
I	33 (44.6)
II	14 (18.9)
III	21 (28.4)
Unknown	6 (8.1)
High-risk cytogenetics, *n* (%)	15 (35.7)^a^
Extramedullary disease, *n* (%)	22 (29.7)
No. of prior lines of therapy, median (range)	3.0 (1–9)
Previous autologous stem cell transplantation, *n* (%)	18 (24.3)
Prior therapies, *n* (%)	
Proteasome inhibitors	54 (73.0)
Bortezomib	53 (71.6)
Carfilzomib	3 (4.1)
Immunomodulatory agents	65 (87.8)
Lenalidomide	35 (47.3)
Pomalidomide	3 (4.1)
Thalidomide	47 (63.5)
Prior proteasome inhibitors + immunomodulatory agents	48 (64.9)
Monoclonal antibodies	2 (2.7)
Daratumumab	1 (1.4)
Isatuximab	1 (1.4)

*ISS* International staging system, *ECOG* Eastern Cooperative Oncology Group

^a^*N* = 42 with available data. High-risk cytogenetic features included *t*(4;14), *t*(14;16), and del(17p)

CAR-T cells were successfully manufactured for all patients and infused at a median dose of 0.513 (range, 0.07–2.10) × 10^6^ cells/kg. At the clinical cutoff date of May 25, 2021, 1 patient was lost to follow-up, 34 patients had died (including 28 due to disease progression), and 39 patients were still on study. The median follow-up was 47.8 months (range, 0.4–60.7 months).

### Long-term safety

Treatment-related safety consists of acute and chronic adverse effects. Within 100 days post-infusion, all patients experienced ≥ 1 acute AEs (Table 2). Grade ≥ 3 AEs occurred in 45 of 74 patients (60.8%): Most common were leukopenia (19/74; 25.7%), thrombocytopenia (14/74; 18.9%), and aspartate aminotransferase elevation (15/74; 20.3%).

**Table 2:** Summary of AEs occurring in ≥ 15% of patients

AE, *n* (%)	*N* = 74	
	Any grade	Grade ≥ 3
Any AE	74 (100)	45 (60.8)
Hematologic		
Leukopenia	32 (43.2)	19 (25.7)
Thrombocytopenia	31 (41.9)	14 (18.9)
Anemia	22 (29.7)	11 (14.9)
Non-hematologic		
Pyrexia	68 (91.9)	11 (14.9)
Increased AST	28 (37.8)	15 (20.3)
Hypotension	14 (18.9)	4 (5.4)
Increased ALT	13 (17.6)	0
Cough	13 (17.6)	1 (1.4)
Cytokine release syndrome	68 (91.9)	7 (9.5)^a^
Neurotoxicity	1	0

*AE* adverse event, *AST* aspartate transaminase, *ALT*, alanine aminotransferase, *CRS* cytokine release syndrome

^a^1 patient died 13 days post-treatment due to grade 5 CRS

CRS occurred in 68 (91.9%) patients. The median time to CRS onset was 9 days (range, 1–19) post-infusion. Most events were mild (grade 1, 47.3%; grade 2, 35.1%). Severe CRS was mostly grade 3 (8.1%), with no grade 4 events and 1 grade 5 event. The median duration of CRS was 9 days (range, 3–57); symptoms resolved in 66 patients but persisted in 2 patients. The major supportive measures to manage CRS included tocilizumab (44.6%), oxygen supply (36.5%), and vasopressor therapy (9.5%).

One patient suffered reversible grade 1 central nervous system (CNS) toxicity manifested by aphasia, agitation, and seizure-like activity. No patients had signs or symptoms of parkinsonism.

B cell aplasia following CAR-T cell therapy was the most common chronic AE. Given a deficiency of antibody-producing B cells, microbial infection was closely monitored. Viral activation occurred in 4 of 74 patients, including 3 patients with herpes zoster virus infection at 7, 26, and 46 months, respectively, and 1 with hepatitis B virus activation at 7 months after infusion. Antiviral drug treatment along with supporting care relieved symptoms and rendered the virus undetectable.

Second primary non-hematological tumors were reported in four cases, without detectable myeloma cells. All were considered unrelated to LCAR-B38M. One patient was diagnosed with lung cancer at 8 months after the CAR-T cell infusion and underwent surgery followed by chemotherapy, but eventually died of pneumonia. One patient was diagnosed with lung cancer at 32 months and survived; one patient developed esophageal cancer at 15 months and ultimately died. Cervical cancer was found in another patient at 8 months, who proceeded with standard anti-tumor treatment and has remained cancer-free. No LCAR-B38M viral integration analysis was conducted in these patients.

Among the 74 patients included in the analysis, 34 deaths were reported, mainly due to disease progression (*n* = 28, including 1 suicide after disease progression). Of the remaining 6 patients, 1 died due to CRS and tumor lysis syndrome (day 13), 1 died due to pulmonary embolism and potential acute coronary syndrome (day 22), 1 died due to respiratory failure associated with subsequent therapy (day 436), 1 died due to esophageal carcinoma (day 493), and 2 died of infection (days 157 and 529).

### Long-term efficacy

**Fig. 1:**
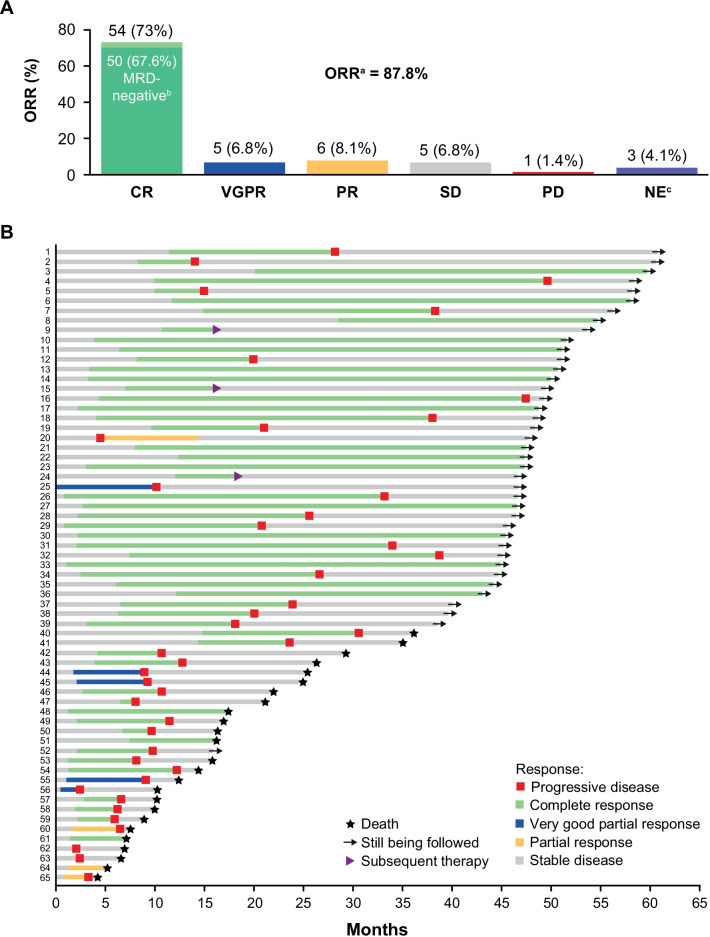
**A** Best response in all patients. **B** Duration of response in patients with a response (≥ PR). *CR* complete response, *MRD* minimal residual disease, *NE* not evaluable, *ORR* overall response rate, *PD* progressive disease, *PR* partial response, *SD* stable disease, *VGPR* very good partial response. ^a^ORR includes patients with ≥ PR. ^b^8-color flow cytometry with cell count up to 500,000 cells. ^c^1 patient died of pulmonary embolism/acute coronary syndrome prior to evaluation, 1 patient died on day 13 due to CRS and TLS, and 1 patient received chemotherapy prior to first assessment and was censored

The median times for initial response and best response were 1.02 months (range, 0.4–3.5) and 3.32 months (range, 0.43–28.52), respectively. At 3 months post-infusion, 70 of 74 patients had survived for evaluation of disease status. ORR was 87.8%; 54 (73%) patients achieved complete response (CR), of which 50 (67.6%) had MRD-negative CR (at 10^–4^). The remainder had very good partial response (VGPR; *n* = 5) or PR (*n* = 6; Fig. 1A). The ORRs and CR rates were comparable between subgroups differentiated by age, time from disease diagnosis to CAR-T cell therapy, disease stage, number of prior therapies, and prior PI/IMiD exposure (Additional file 1: Tables S1 and S2). Patients with high-risk cytogenetics had comparable CR rates to those with standard risk (80% vs. 66.7%), and no statistically significant differences in survival outcomes (Additional file 1: Fig. S2 A–C).

Overall, the median DOR reached 23.26 months (95% CI 13.04–32.69). The median DOR for patients with CR (29.14 months) was longer than patients with < CR (5.03 months). Patients with EMD at baseline had a shorter DOR, although they had a similar time to response as those without EMD [19].

**Fig. 2:**
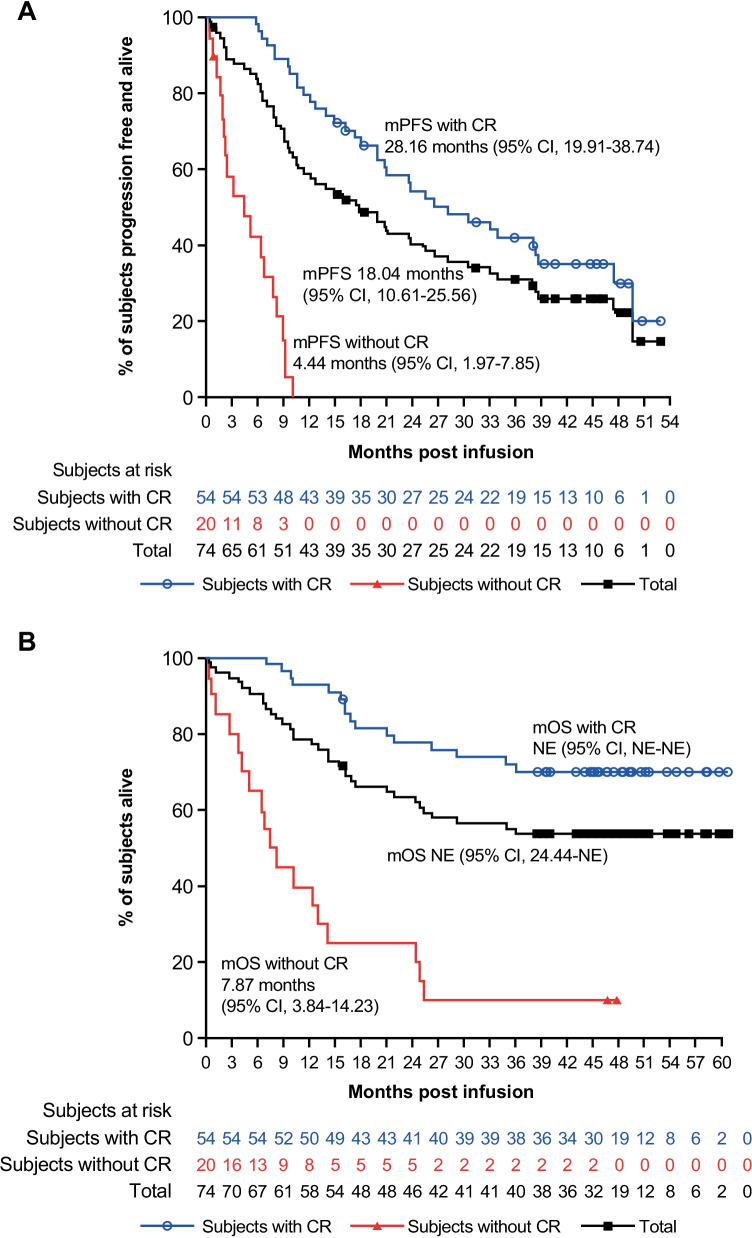
**A** Progression-free survival and **B** overall survival by CR. *CI* confidence interval, *CR* complete response, *NE* not evaluable, *OS* overall survival, *PFS* progression-free survival

Of the 39 patients still alive at clinical cutoff, 16 had ongoing CR without disease progression (Fig. 1B). The median PFS was 18.04 months for all patients and longer in patients with CR (median 28.16 months) than those not achieving CR (median 4.44 months; Fig. 2A). The longest disease-free survival without maintenance treatment was 60 months, observed in a patient with *λ* light-chain-type MM, who had extensive EMD.

Median OS was not yet reached (Fig. 2B). The 24-month OS rate was 63.4% for all patients and 77.5% for patients with CR. In contrast, the 24-month OS rate was only 25.0% for those with < CR.

Of 65 patients achieving ≥ PR, 43 subsequently developed progressive disease (PD), mostly in the first 24 months (32 patients). The median OS of patients with PD was 36.14 (95% CI 21.13–NE) months; 22 patients with PD died. The last death occurred at 36.14 months of follow-up. Notably, 21 patients with PD survived, some of whom were re-treated with a PI, entered another CAR-T clinical study, or proceeded with a previously unused drug. After receiving the next line of treatment, the median OS for these 21 patients with PD was 19.68 months, which was significantly longer than that for patients with PD reported in the historical literature [20].

Although the Kaplan–Meier PFS curve showed that the patients continued to progress with longer follow-up, the OS curve started to stabilize after 24 months, and no patient died beyond 36 months. For patients who had progressed beyond 24 months, several achieved response in subsequent therapy, even with agents used previously.

### Biomarkers and their clinical relevance

**Fig. 3:**
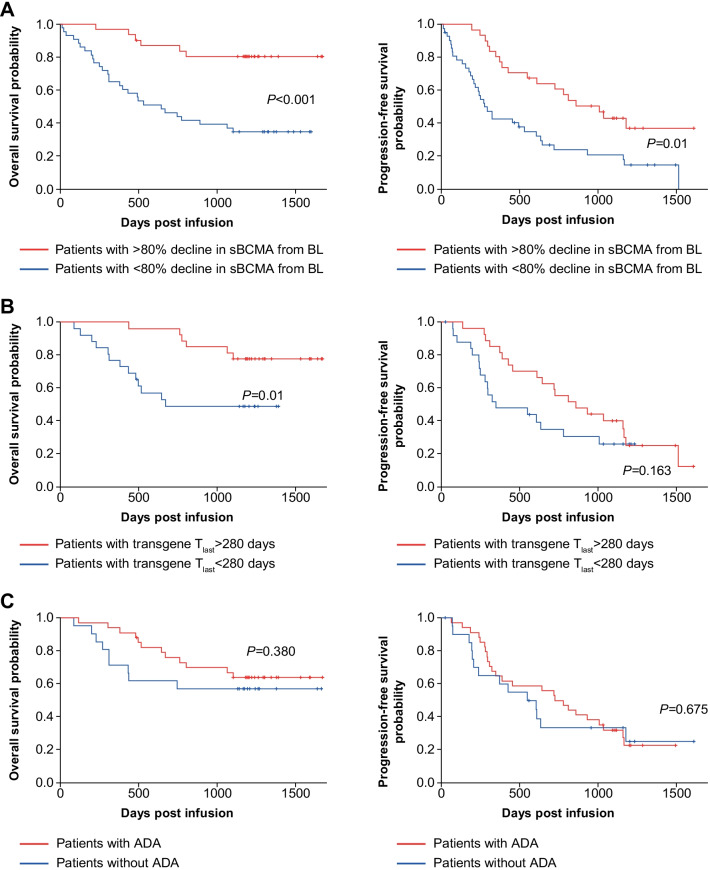
Overall survival (left) and progression-free survival (right) by **A** sBCMA decline, **B** transgene persistence, and **C** ADA. 280 days is the median T_last_ value for the patient population. *ADA* antidrug antibody, *BL* baseline, *sBCMA* soluble B cell maturation antigen, *T*_*last*_ last time point of detectable transgene

Serum sBCMA was detected in all 47 patients with evaluable specimens. Thirty-seven patients had a remarkable decrease in sBCMA (mean, −94.2% relative to baseline; Additional file 1: Table S3). The median time to nadir sBCMA was 155 days (range, 3–1444 days). Of the 11 evaluable patients at relapse (within 7 days before or within 2 days after relapse), sBCMA levels rebounded much higher (median 38,909 pg/mL) than in the 14 evaluable non-relapse patients (median 17,357.5 pg/mL, *P* = 0.002). Patients with > 80% decline of sBCMA from baseline had a longer OS (*P* < 0.001) and PFS (*P* = 0.01) than those with a mild decrease in sBCMA (Fig. 3A). Patients with CR had a sharper drop in sBCMA level from baseline than patients who did not achieve CR, but this did not reach statistical significance (Additional file 1: Table S4). No correlation between sBCMA and DOR was observed (Additional file 1: Fig. S3A).

Duration of CAR transgene persistence (*T*_last_) varied in individuals (median 280 days; range, 8–1466). We assessed the possible impact of the lymphodepletion regimen and PD on transgene persistence, but no significant difference was detected in the Cyc + Flu combined group (*n* = 7) versus the cyclophosphamide alone group (*n* = 46; *P* = 0.937), acknowledging the limitation of this analysis due to imbalance in sample sizes. Nor was there a difference in transgene persistence in patients with PD versus those without PD (287.5 vs. 231 days, *P* = 0.921, Additional file 1: Table S5). However, patients with *T*_last_ > 280 days had a prolonged OS than those with *T*_last_ < 280 days (*P* = 0.010); however, this correlation was not observed with PFS or DOR (Fig. 3B, Additional file 1: Fig. S3B).

ADAs were measured in 55 evaluable patient specimens, and 34 patients (62%) tested positive. The mean time to ADA positivity was 229.6 days (range, 13–905 days). Notably, the appearance of ADAs was less frequent in the Cyc + Flu group than cyclophosphamide alone (28.6% vs. 66.7%). There was no significant difference in ADA positivity between single-dose infusion (6/9, 66.7%) and split infusion (28/46, 60.9%). Patients positive for ADAs (*n* = 32) showed no difference in transgene persistence than those negative for ADAs (*n* = 18) (median 339 vs. 137 days; *P* = 0.104; Additional file 1: Table S5). The existence of ADAs also had no impact on OS, PFS, or DOR (Fig. 3C, Additional file 1: Fig. S3C). However, the assay used to measure ADAs for this analysis was exploratory in nature and more data are needed to validate these findings.

**Fig. 4:**
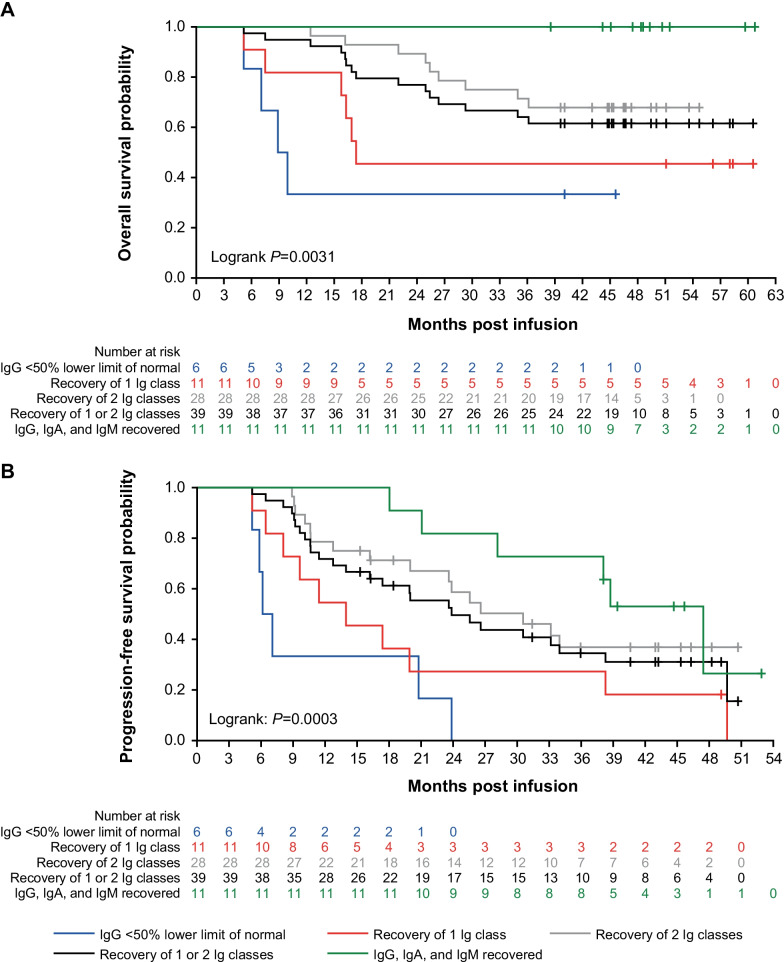
**A** Overall survival and **B** progression-free survival by hypogammaglobulinemia resolving status in patients with PR or better. *Ig* Immunoglobulin, *PR* partial response

The recovery of immunoglobulin (Ig) was investigated in the patients who achieved ≥ PR. The median time to recovery was 30 months for serum IgG, > 24 months for IgA, and > 9 months for IgM (Additional file 1: Table S6). Patients with a full restoration of immunoglobulin had longer OS and PFS than those without a complete recovery (*P* = 0.0031 and 0.0003, respectively; Fig. 4). Patients with IgG < 50%, the lower limit of normal, had a worse prognosis than those having a higher IgG level, indicating that immunoglobulin recovery may be a prognostic predictor.

### Extramedullary disease

EMD represents a high-risk clinical indicator associated with poor prognosis [21, 22]. Of 74 patients in the study, 22 patients (Xi’an site, *n* = 17; East China, *n* = 5) had baseline EMD, including EMD not adjacent to bone (*n* = 15; 2 of whom had concomitant plasma cell leukemia) and bone-based (*n* = 7). No statistical differences in gender, age, or number of prior lines of therapy were apparent in patients with EMD versus those without EMD. However, the light-chain MM subtype was more frequent in patients with EMD than the IgG or IgA subtypes. As expected, despite a similar ORR in the EMD and the non-EMD subgroup (81.8% vs. 90.4%), the EMD subgroup demonstrated a lower CR rate than the non-EMD subgroup (54.5% vs. 80.8%). Median PFS was shorter in patients with EMD [8.9 months (95% CI 5.85–10.61) vs. 26.58 months (95% CI 56.8–81.5; *P* < 0.001)]. Likewise, the median OS of the EMD subgroup was 14.28 months (95% CI 8.87–29.27) whereas the median OS of the non-EMD subgroup was not reached. Of the 22 patients with baseline EMD, 18 showed extramedullary relapse, 1 died due to infection, 1 was nonresponsive to LCAR-B38M, and 2 patients have not relapsed. EMD was a poor prognostic factor in this study, consistent with the literature [23].

## Discussion

This report represents the longest follow-up to date for any CAR-T therapy in patients with MM. The key findings in the LEGEND-2 trial are largely consistent with the results generated in the CARTITUDE-1 study [14], despite population differences between the two studies. Owing to drug availability and accessibility in China, the number and types of prior lines of treatment differed from that of CARTITUDE-1, including few patients with exposure to monoclonal antibodies or carfilzomib and relatively few with autologous stem cell transplantation despite younger age. Chinese patients also have more aggressive disease than Western populations [24, 25], including more patients in LEGEND-2 with ISS Stage III disease (28% vs. 14%) and EMD (30% vs. 13%). However, the LEGEND-2 data suggest that LCAR-B38M could be an important treatment option for these patients with unprecedented efficacy and a positive benefit/risk profile.

Over 4 years, LCAR-B38M exerted a strong anti-myeloma activity with an ORR of 87.8%, MRD negativity rate of 67.6%, and a median PFS of 18 months in RRMM. Interestingly, although the PFS curve continuously dropped over time, the OS curve started to plateau, indicating that patients who had later progression responded well to subsequent therapy. PFS in LEGEND-2 was shorter than in CARTITUDE-1, which is likely attributable to more aggressive disease in the LEGEND-2 population. Additionally, some patients in LEGEND-2 received a below-target dose of LCAR-B38M, whereas all CARTITUDE-1 patients received a target dose. The PFS results are nonetheless positive in Chinese patients and were achieved with a low dose of CAR-T cells (median 0.51 × 10^6^ cells/kg).

In comparison, the pivotal trial of idecabtagene vicleucel (ide-cel) showed an ORR of 73%, MRD negativity rate of 26%, and a median PFS of 8.8 months in patients with heavily pretreated RRMM [26]. Belantamab mafodotin, the US FDA-approved, first-in-class antibody–drug conjugate targeting BCMA, demonstrated an ORR of 31% and a median PFS of 2.9 months at the approved dose [27]. Selinexor, the first orally selective inhibitor of nuclear export compound, showed a 26% ≥ PR rate and a median PFS of 3.7 months in combination with dexamethasone [28]. A possible reason for a strong therapeutic effect on malignant plasma cells of LCAR-B38M might lie in the specially designed CAR construct featuring two tandemly arranged BCMA-targeting, llama-derived heavy-chain domains, which is structurally distinct from other anti-BCMA CAR-T cell products [29, 30].

In addition to favorable efficacy, LCAR-B38M showed a manageable safety profile. Hematologic AEs were the most commonly reported. CRS was reported in most (92%) patients, but was mostly grade 1 or 2. Nearly all patients (66 of 68) recovered from CRS after anti-IL6 receptor administration and supportive measures; only 1 died due to CRS in the context of tumor lysis syndrome. The median time to CRS onset (9 days) is consistent with that observed in the CARTITUDE-1 study (7 days) but longer than with ide-cel treatment (1 day) [26]. Such a delay might be attributable to the split infusion and/or a low weight-based dose. Like other CAR-T studies conducted in China, neurological toxicities were very uncommon, with only one grade 1 CNS toxicity reported. Severe neurotoxicity caused by LCAR-B38M was comparable or lower than that of other engineered products in the same class [8, 26, 29, 30]. The frequency of SPM was 5.4%, consistent with literature reports of patients previously treated with lenalidomide [31].

We investigated the relationships between several biomarkers with CAR-T dosing regimen, lymphodepletion regimen, and survival outcomes. Preliminary data on ADA showed that although the infusion regimen (single vs. split) did not impact ADA production, patients with a Cyc + Flu combination lymphodepletion regimen were less likely to develop ADAs than those lymphodepleted with cyclophosphamide alone. Nevertheless, the presence of ADAs had no impact on DOR, PFS, or OS. The persistence of CAR-T cells was another index that was closely monitored. Neither dose-splitting nor lymphodepletion regimens influenced the persistence of CAR-T cells, and persistence did not differ in patients with or without PD. However, patients with longer CAR-T cell persistence had prolonged OS. Finally, a rapid reduction in sBCMA levels could predict longer OS and PFS, while a significant rebound of sBCMA corresponded to disease relapse. An important question for future research is that of BCMA allelic loss at baseline in the Chinese RRMM population and after anti-BCMA CAR-T therapy.

It was noteworthy that the median survival time after PD post-LCAR-B38M treatment was significantly longer than that of newly approved therapies, as reflected in the plateauing of the OS curve but not the PFS curve [2, 3]. It is speculated that CAR-T therapy may help re-sensitize myeloma cells to previously used drugs for patients who progressed > 2 years after CAR-T cell treatment. The mechanisms for this remain unknown; however, it is possible that LCAR-B38M therapy imparts long-term changes to the immune/tumor microenvironment and/or the genetic profile or clonal distribution of the myeloma cells. Alternatively, prolonged survival could be related to the array of therapeutic options available to which patients had no previous exposure, including newer PIs and IMiDs, and mAbs that were not available before the study. For example, 6 patients opted for anti-CD38 treatment after relapse and subsequently achieved remission. Nevertheless, the efficacy of LCAR-B38M in this less heavily treated, albeit more ill, population of patients compared with CARTITUDE-1 suggests that it will be efficacious as earlier-line therapy. This is being formally tested in the ongoing phase 3, randomized, active comparator CARTITUDE-4 (NCT04181827), CARTITUDE-5 (NCT04923893), and CARTITUDE-6 (NCT05257083) studies.

EMD still constituted a key cause of MM recurrence in this study. Extramedullary plasmablasts have been long considered highly invasive and resistant to conventional treatment. Patients with EMD usually lose response in a relatively shorter time compared with those without EMD. In this study, LCAR-B38M had a significant therapeutic effect in RRMM patients with EMD, reflecting its potent killing capacity against therapy-resistant extramedullary plasmablasts. A novel modality to consolidate and maintain the curative effect of CAR-T therapy is warranted [32].

## Conclusions

The 4-year follow-up of the LEGEND-2 study provides evidence of long-term efficacy and safety of LCAR-B38M in the treatment of RRMM in China. Cilta-cel, using an identical construct to LCAR-B38M, has been evaluated in the CARTITUDE-1 study with distinct ethnic groups in the USA and has shown unprecedented efficacy [14]; and an ongoing study in China, with an almost identical design, aims to further confirm the results of CARTITUDE-1 in a Chinese population (CARTIFAN-1; NCT03758417).

## Supplementary Information

**Additional file1: Fig. S1.** Study Design. **Fig. S2.** **A** Duration of response, **B** overall survival, and **C** progression-free survival by cytogenetic risk. **Fig. S3.** Relationship of duration of response with **A** sBCMA, **B** transgene persistence, and **C** antidrug antibodies. **Table S1.** Best response by subgroup. **Table S2.** Best response by prior therapy. **Table S3.** sBCMA levels. **Table S4.** sBCMA levels by response. **Table S5.** Transgene persistence by lymphodepletion regimen, PD, and ADA. **Table S6.** Immunoglobulin recovery over time.

## Data Availability

Access to study data may be requested from the corresponding authors.

## Abbreviations

AE: Adverse event
ADA: Antidrug antibody
BCMA: B cell maturation antigen
CAR: Chimeric antigen receptor
Cilta-cel: Ciltacabtagene autoleucel
CNS: Central nervous system
CR: Complete response
CI: Confidence interval
Cyc + Flu: Cyclophosphamide plus fludarabine
CRS: Cytokine release syndrome
DOR: Duration of response
EMD: Extramedullary disease
Ide-cel: Idecabtagene vicleucel
Ig: Immunoglobulin
MRD: Minimal residual disease
MM: Multiple myeloma
ORR: Overall response rate
OS: Overall survival
PR: Partial response
PFS: Progression-free survival
PD: Progressive disease
PI: Proteasome inhibitor
IMiD: Immunomodulatory drug
RR: Relapsed/refractory
sBCMA: Soluble B cell maturation antigen
*T*_last_: Last time point of detectable transgene
VGPR: Very good partial response
