# Supplementary material for: Four-year follow-up of LCAR-B38M in relapsed or refractory multiple myeloma: a phase 1, single-arm, open-label, multicenter study in China (LEGEND-2)
Source: J Hematol Oncol. 2022 Jul 6;15:86. doi: 10.1186/s13045-022-01301-8 (PMC9261106; doi:10.1186/s13045-022-01301-8)

**Supplementary Information**

**Supplementary Table 1.** Best Response by Subgroup

|  | **Age Group** | | **Time Since Initial Diagnosis** | | **ISS Stage^b^** | |
| --- | --- | --- | --- | --- | --- | --- |
| **n (%)** | **≤54 years**  **(n=37)** | **>54 years**  **(n=37)** | **≤4 years**  **(n=50)** | **>4 years**  **(n=24)** | **III**  **(n=21)** | **I/II**  **(n=47)** |
| Overall response | 33 (89.2%) | 32 (86.5%) | 43 (86.0%) | 22 (91.7%) | 18 (85.7%) | 42 (89.4%) |
| CR | 28 (75.7%) | 26 (70.3%) | 35 (70.0%) | 19 (79.2%) | 15 (71.4%) | 35 (74.5%) |
| MRD-negative^a^ | 27 (73.0%) | 23 (62.2%) | 32 (64.0%) | 18 (75.0%) | 14 (66.7%) | 33 (70.2%) |
| VGPR | 2 (5.4%) | 3 (8.1%) | 4 (8.0%) | 1 (4.2%) | 0 | 5 (10.6%) |
| PR | 3 (8.1%) | 3 (8.1%) | 4 (8.0%) | 2 (8.3%) | 3 (14.3%) | 2 (4.3%) |
| SD | 2 (5.4%) | 3 (8.1%) | 4 (8.0%) | 1 (4.2%) | 1 (4.8%) | 3 (6.4%) |
| PD | 0 | 1 (2.7%) | 1 (2.0%) | 0 | 1 (4.8%) | 0 |
| NE | 2 (5.4%) | 1 (2.7%) | 2 (4.0%) | 1 (4.2%) | 1 (4.8%) | 2 (4.3%) |
| ≥VGPR | 30 (81.1%) | 29 (78.4%) | 39 (78.0%) | 20 (83.3%) | 15 (71.4%) | 40 (85.1%) |

^a^MRD status was based on 8-color flow cytometry.

^b^Patients with unkown ISS stage are not included.

**Supplementary Table 2.** Best Response by Prior Therapy

|  | **Prior PI and IMiD** | | **≥3 Prior Lines** | | **Prior PI and Prior IMiD and**  **≥3 Prior Lines^a^** | |
| --- | --- | --- | --- | --- | --- | --- |
| **n (%)** | **Yes**  **(n=48)** | **No**  **(n=26)** | **Yes**  **(n=45)** | **No**  **(n=29)** | **Yes**  **(n=35)** | **No**  **(n=39)** |
| Overall response | 41 (85.4%) | 24 (92.3%) | 37 (82.2%) | 28 (96.6%) | 29 (82.9%) | 36 (92.3%) |
| CR | 35 (72.9%) | 19 (73.1%) | 31 (68.9%) | 23 (79.3%) | 25 (71.4%) | 29 (74.4%) |
| VGPR | 5 (10.4%) | 0 | 4 (8.9%) | 1 (3.4%) | 4 (11.4%) | 1 (2.6%) |
| PR | 1 (2.1%) | 5 (19.2%) | 2 (4.4%) | 4 (13.8%) | 0 | 6 (15.4%) |

^a^Subjects treated with prior lines>=3 which included both PI and IMiD.

**Supplementary Table 3.** sBCMA Levels

| **n (%)** | **All Patients^a^**  **(N=60)** |
| --- | --- |
| Baseline sBCMA – median (range) pg/mL | 141,676^b^  (7066–341,476) |
| Nadir sBCMA – median (range) pg/mL | 10,158  (274–383,541) |
| Change from baseline, % | –94%^c^ |
| Time to nadir sBCMA – median (range) days | 155 (3–1444) |

^a^With available sBCMA data.

^b^N=48.

^c^N=47.

**Supplementary Table 4.** sBCMA Levels by Response

| **sBCMA Change From Baseline (%)** | **Patients With CR**  **(N=37)** | **Patients without CR**  **(N=10)** |
| --- | --- | --- |
| Mean (SD) | -56.3 (180.37) | -45.9 (46.98) |
| *P* value | 0.099 | |
| Median (range) | -94.2  (-99.8–991.1) | -38.4  (-99.6–12.3) |

**Supplementary Table 5.** Transgene Persistence by Lymphodepletion Regimen, PD, and ADA

|  | **Lymphodepletion Regimen** | | **PD** | | **ADA** | |
| --- | --- | --- | --- | --- | --- | --- |
|  | **Cyc+Flu**  **(n=7)** | **Cyc**  **(n=46)** | **Yes**  **(n=38)** | **No**  **(n=15)** | **Positive**  **(n=32)** | **Negative**  **(n=18)** |
| T_last_ – median (range) days | 314  (31–1326) | 261  (8–1037) | 288  (10–1016) | 231  (8–1466) | 339  (12–1466) | 137  (8–1016) |
| *P* value | 0.937 | | 0.921 | | 0.104 | |

ADA, anti-drug antibodies; Cyc, cyclophosphamide; Flu, fludarabine; PD, progressive disease.

**Supplementary Table 6.** Immunoglobulin Recovery Over Time

|  | **IgA** | | **IgG** | | **IgM** | |
| --- | --- | --- | --- | --- | --- | --- |
| **Month post-infusion** | **N** | **Median change from baseline, g/L** | **N** | **Median change from baseline, g/L** | **N** | **Median change from baseline, g/L** |
| Month 3 | 28 | -0.489 | 28 | -1.775 | 28 | 0.000 |
| Month 6 | 29 | -0.440 | 29 | -1.150 | 28 | 0.118 |
| Month 9 | 27 | -0.226 | 28 | -1.065 | 28 | **0.178** |
| Month 12 | 32 | -0.005 | 32 | -0.452 | 32 | 0.408 |
| Month 18 | 30 | 0.083 | 31 | -9.180 | 31 | 0.481 |
| Month 24 | 25 | **0.384** | 25 | -8.500 | 25 | 0.706 |
| Month 30 | 16 | 0.291 | 17 | **0.640** | 17 | 0.510 |
| Month 36 | 18 | 0.559 | 18 | -8.135 | 18 | 0.576 |
| Month 42 | 12 | 0.524 | 12 | -8.255 | 12 | 0.822 |
| Month 48 | 10 | -0.375 | 10 | -3.100 | 10 | 0.387 |
| Month 54 | 3 | 0.919 | 3 | -7.850 | 3 | 0.615 |

Bolded values show median timepoint of recovery.

**Supplementary Fig. 1.** Study Design


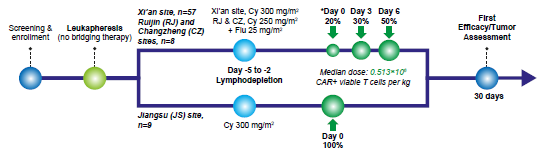


**Supplementary Fig. 2 A** Duration of response, **B** overall survival, and **C** progression-free survival by cytogenetic risk


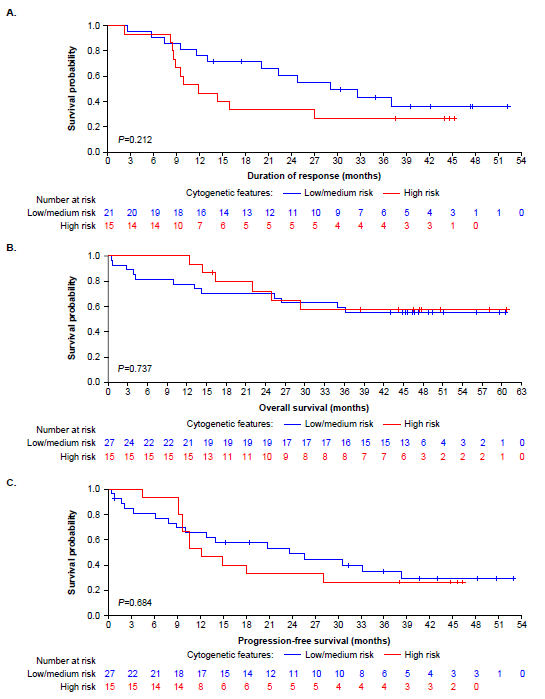


**Supplementary Fig. 3** Relationship of duration of response with **A** sBCMA, **B** transgene persistence, and **C** anti-drug antibodies


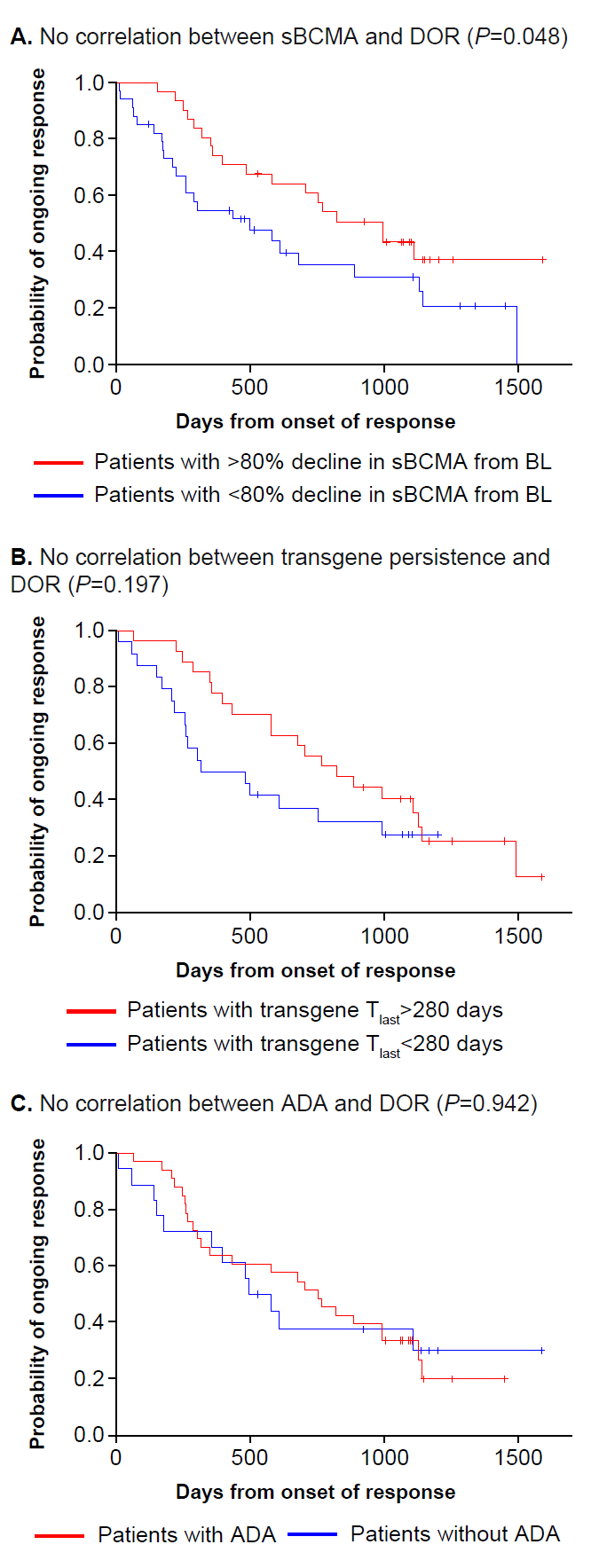

Supplement: Supplementary file 1 — Additional file1: Fig. S1. Study Design. Fig. S2. A Duration of response, B overall survival, and C progression-free survival by cytogenetic risk. Fig. S3. Relationship of duration of response with A sBCMA, B transgene persistence, and C antidrug antibodies. Table S1. Best response by subgroup. Table S2. Best response by prior therapy. Table S3. sBCMA levels. Table S4. sBCMA levels by response. Table S5. Transgene persistence by lymphodepletion regimen, PD, and ADA. Table S6. Immunoglobulin recovery over time. [file 13045_2022_1301_MOESM1_ESM.docx]
